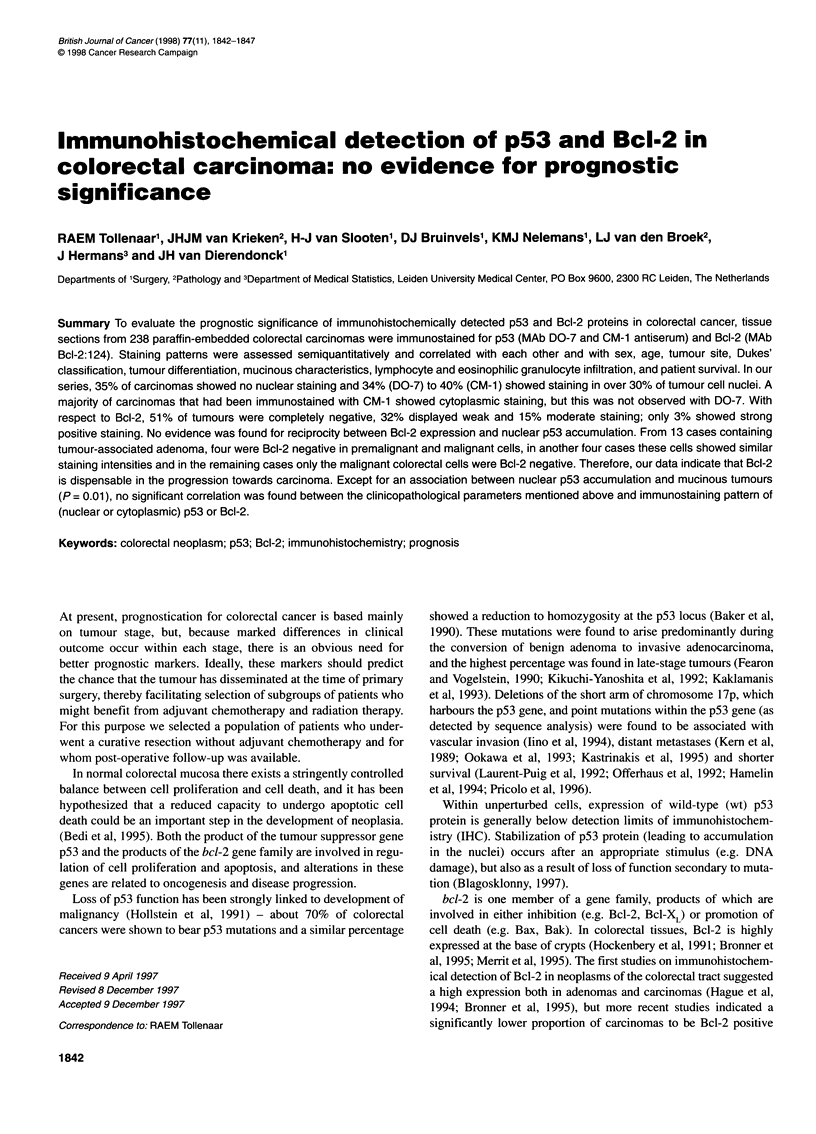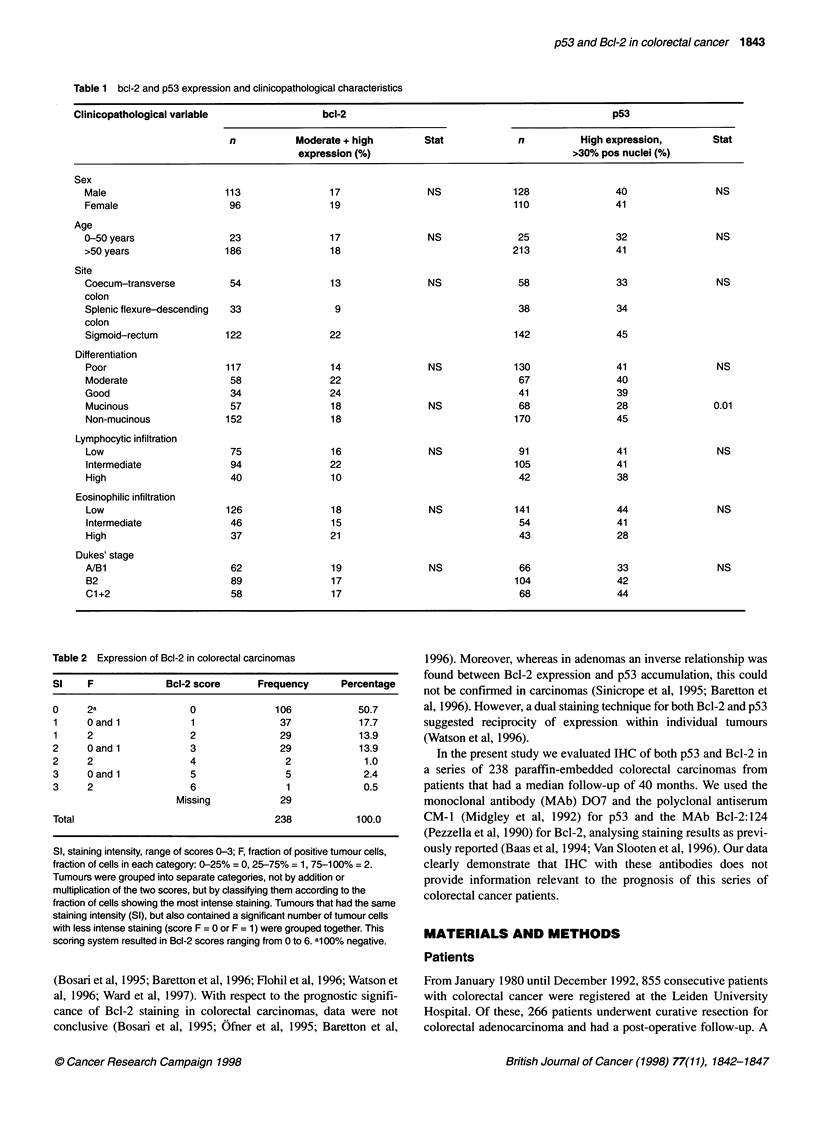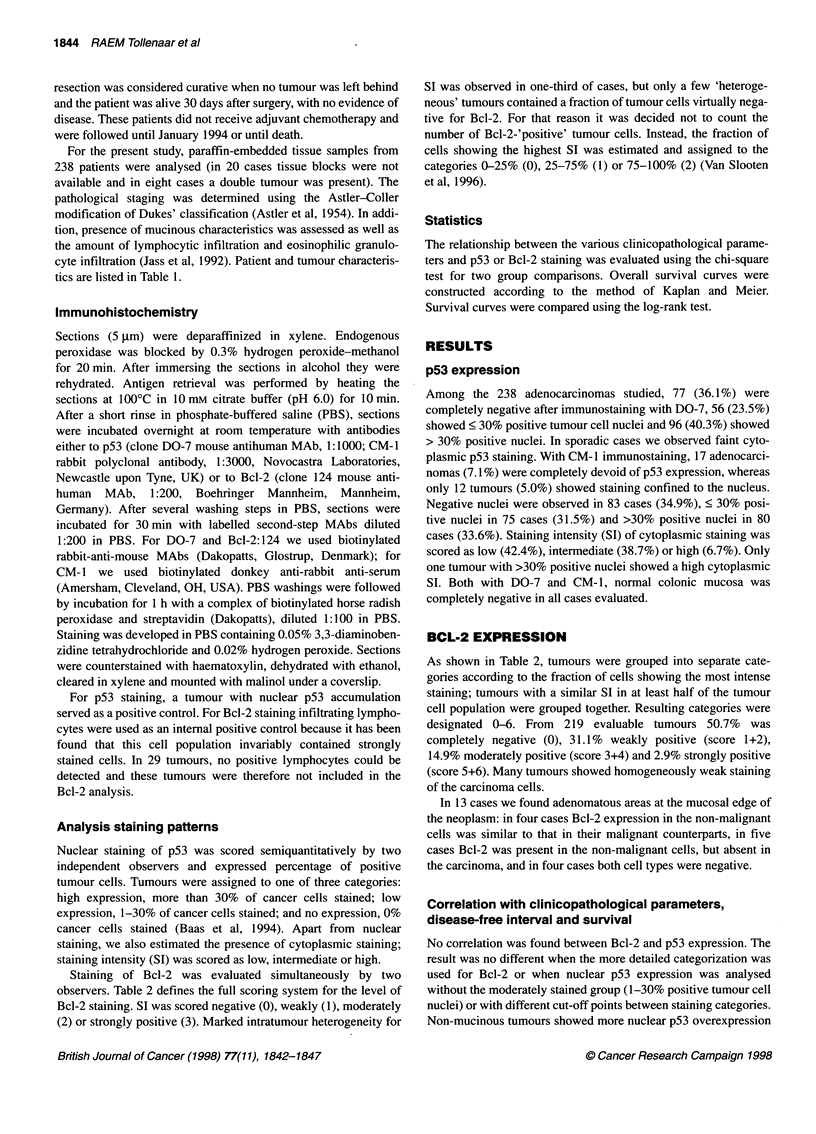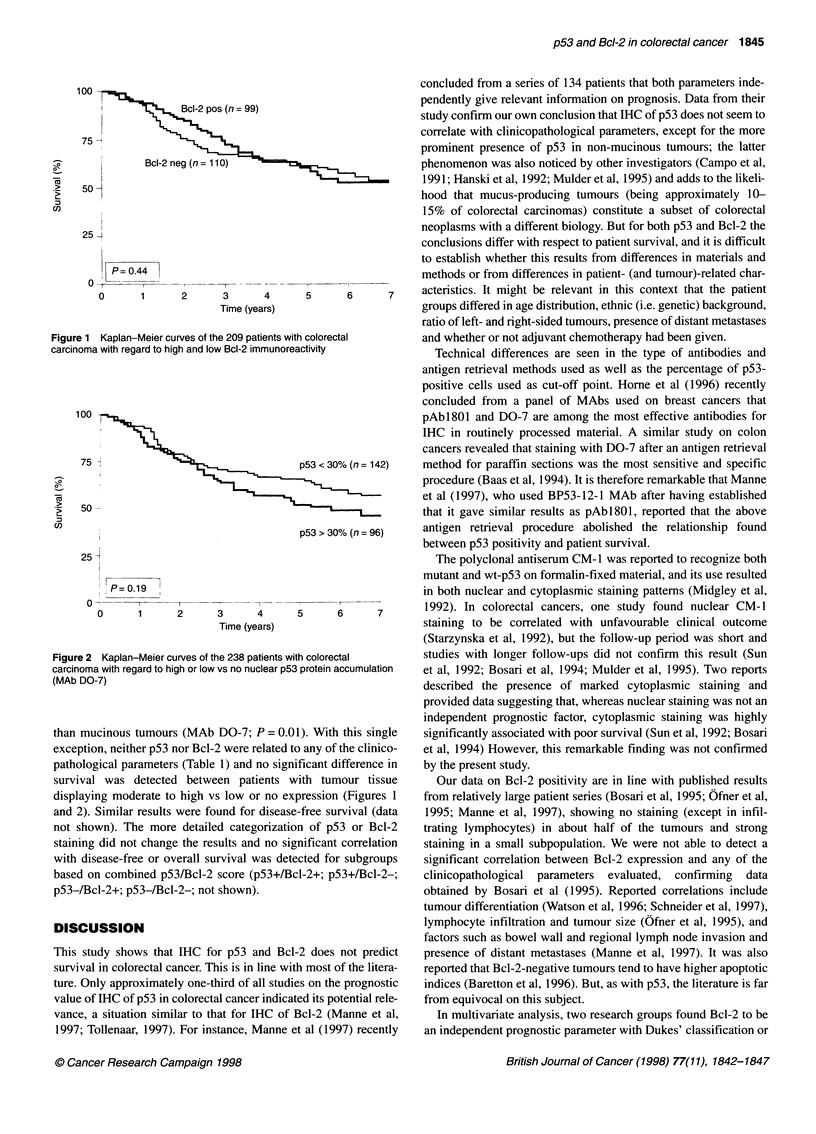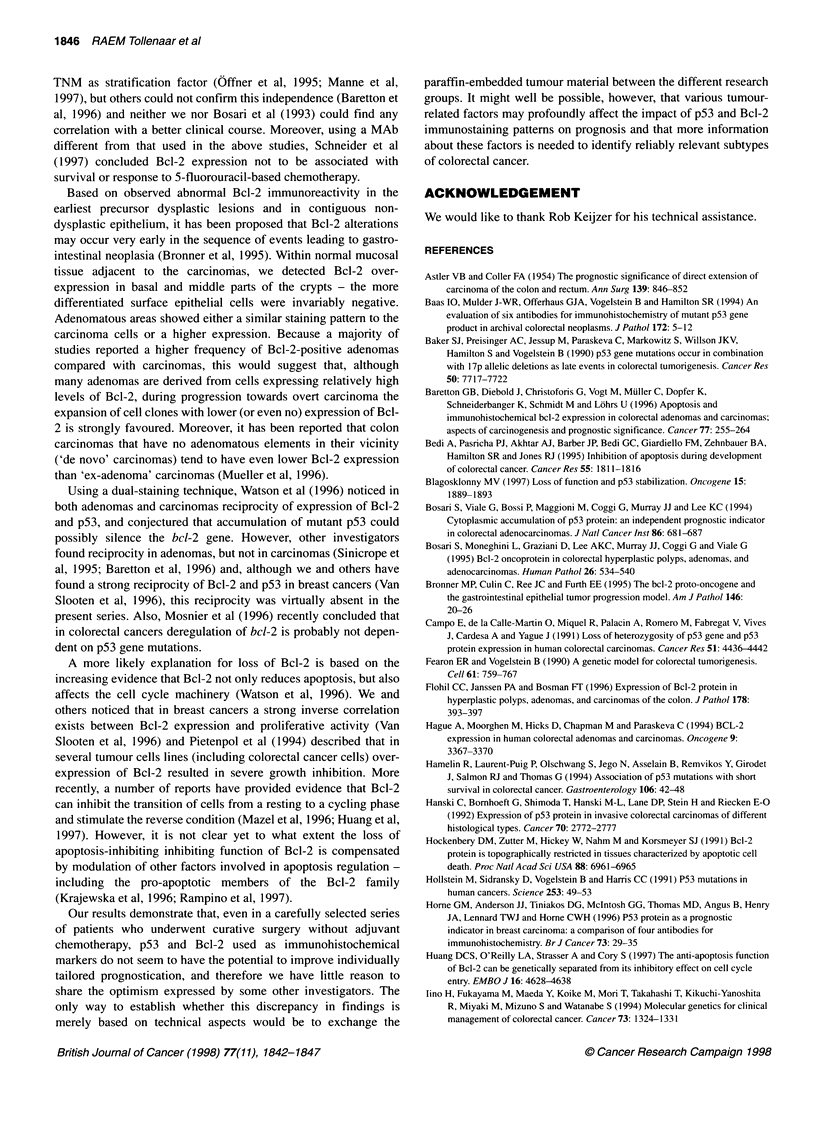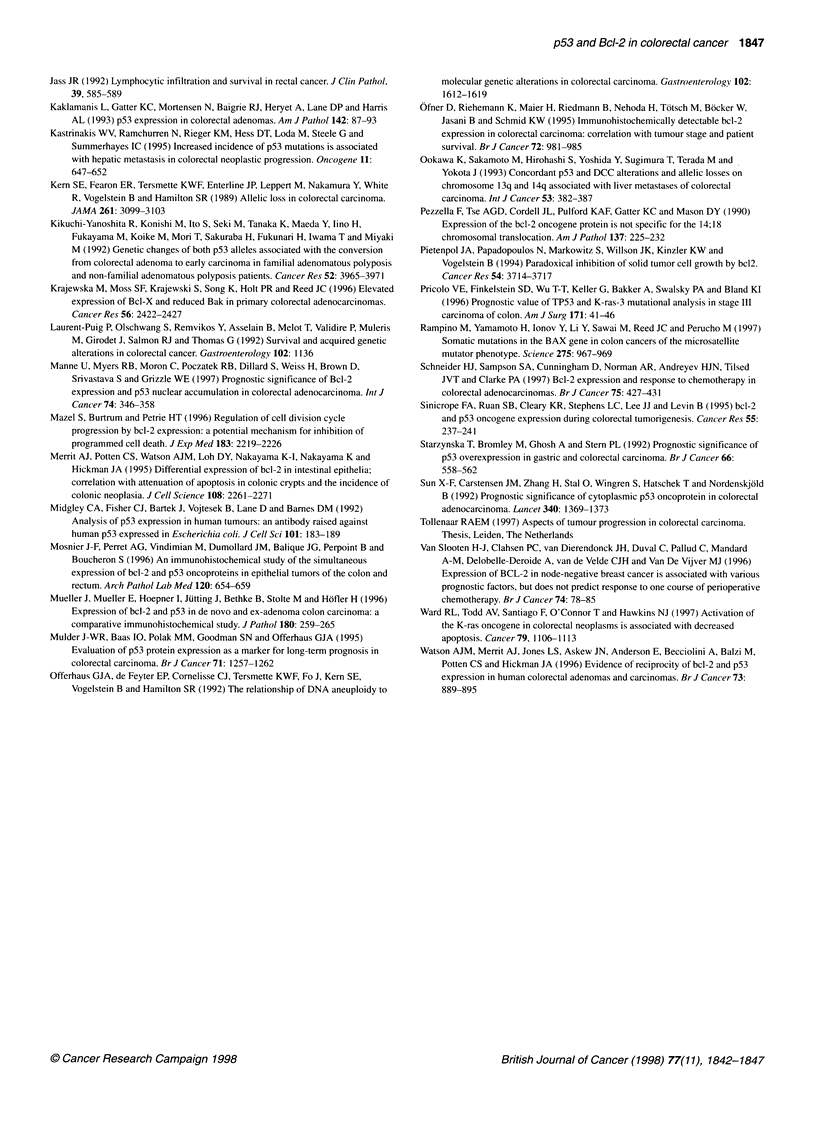# Immunohistochemical detection of p53 and Bcl-2 in colorectal carcinoma: no evidence for prognostic significance.

**DOI:** 10.1038/bjc.1998.306

**Published:** 1998-06

**Authors:** R. A. Tollenaar, J. H. van Krieken, H. J. van Slooten, D. J. Bruinvels, K. M. Nelemans, L. J. van den Broek, J. Hermans, J. H. van Dierendonck

**Affiliations:** Department of Surgery, Leiden University Medical Center, The Netherlands.

## Abstract

To evaluate the prognostic significance of immunohistochemically detected p53 and Bcl-2 proteins in colorectal cancer, tissue sections from 238 paraffin-embedded colorectal carcinomas were immunostained for p53 (MAb DO-7 and CM-1 antiserum) and Bcl-2 (MAb Bcl-2:124). Staining patterns were assessed semiquantitatively and correlated with each other and with sex, age, tumour site, Dukes' classification, tumour differentiation, mucinous characteristics, lymphocyte and eosinophilic granulocyte infiltration, and patient survival. In our series, 35% of carcinomas showed no nuclear staining and 34% (DO-7) to 40% (CM-1) showed staining in over 30% of tumour cell nuclei. A majority of carcinomas that had been immunostained with CM-1 showed cytoplasmic staining, but this was not observed with DO-7. With respect to Bcl-2, 51% of tumours were completely negative, 32% displayed weak and 15% moderate staining; only 3% showed strong positive staining. No evidence was found for reciprocity between Bcl-2 expression and nuclear p53 accumulation. From 13 cases containing tumour-associated adenoma, four were Bcl-2 negative in premalignant and malignant cells, in another four cases these cells showed similar staining intensities and in the remaining cases only the malignant colorectal cells were Bcl-2 negative. Therefore, our data indicate that Bcl-2 is dispensable in the progression towards carcinoma. Except for an association between nuclear p53 accumulation and mucinous tumours (P = 0.01), no significant correlation was found between the clinicopathological parameters mentioned above and immunostaining pattern of (nuclear or cytoplasmic) p53 or Bcl-2.


British Journal of Cancer (1998) 77(11), 1842–1847
© 1998 Cancer Research Campaign

### Immunohistochemical detection of p53 and Bcl-2 in colorectal carcinoma: no evidence for prognostic significance

**RAEM Tollenaar[1], JHJM van Krieken[2], H-J van Slooten[1], DJ Bruinvels[1], KMJ Nelemans[1], LJ van den Broek[2], J Hermans[3] and JH van Dierendonck[1]**

Departments of [1]Surgery, [2]Pathology and [3]Department of Medical Statistics, Leiden University Medical Center, PO Box 9600, 2300 RC Leiden, The Netherlands

**Summary** To evaluate the prognostic significance of immunohistochemically detected p53 and Bcl-2 proteins in colorectal cancer, tissue sections from 238 paraffin-embedded colorectal carcinomas were immunostained for p53 (MAb DO-7 and CM-1 antiserum) and Bcl-2 (MAb Bcl-2:124). Staining patterns were assessed semiquantitatively and correlated with each other and with sex, age, tumour site, Dukes' classification, tumour differentiation, mucinous characteristics, lymphocyte and eosinophilic granulocyte infiltration, and patient survival. In our series, 35% of carcinomas showed no nuclear staining and 34% (DO-7) to 40% (CM-1) showed staining in over 30% of tumour cell nuclei. A majority of carcinomas that had been immunostained with CM-1 showed cytoplasmic staining, but this was not observed with DO-7. With respect to Bcl-2, 51% of tumours were completely negative, 32% displayed weak and 15% moderate staining; only 3% showed strong positive staining. No evidence was found for reciprocity between Bcl-2 expression and nuclear p53 accumulation. From 13 cases containing tumour-associated adenoma, four were Bcl-2 negative in premalignant and malignant cells, in another four cases these cells showed similar staining intensities and in the remaining cases only the malignant colorectal cells were Bcl-2 negative. Therefore, our data indicate that Bcl-2 is dispensable in the progression towards carcinoma. Except for an association between nuclear p53 accumulation and mucinous tumours ($P = 0.01$), no significant correlation was found between the clinicopathological parameters mentioned above and immunostaining pattern of (nuclear or cytoplasmic) p53 or Bcl-2.

**Keywords:** colorectal neoplasm; p53; Bcl-2; immunohistochemistry; prognosis

At present, prognostication for colorectal cancer is based mainly on tumour stage, but, because marked differences in clinical outcome occur within each stage, there is an obvious need for better prognostic markers. Ideally, these markers should predict the chance that the tumour has disseminated at the time of primary surgery, thereby facilitating selection of subgroups of patients who might benefit from adjuvant chemotherapy and radiation therapy. For this purpose we selected a population of patients who underwent a curative resection without adjuvant chemotherapy and for whom post-operative follow-up was available.

In normal colorectal mucosa there exists a stringently controlled balance between cell proliferation and cell death, and it has been hypothesized that a reduced capacity to undergo apoptotic cell death could be an important step in the development of neoplasia. (Bedi et al, 1995). Both the product of the tumour suppressor gene p53 and the products of the *bcl*-2 gene family are involved in regulation of cell proliferation and apoptosis, and alterations in these genes are related to oncogenesis and disease progression.

Loss of p53 function has been strongly linked to development of malignancy (Hollstein et al, 1991) – about 70% of colorectal cancers were shown to bear p53 mutations and a similar percentage showed a reduction to homozygosity at the p53 locus (Baker et al, 1990). These mutations were found to arise predominantly during the conversion of benign adenoma to invasive adenocarcinoma, and the highest percentage was found in late-stage tumours (Fearon and Vogelstein, 1990; Kikuchi-Yanoshita et al, 1992; Kaklamanis et al, 1993). Deletions of the short arm of chromosome 17p, which harbours the p53 gene, and point mutations within the p53 gene (as detected by sequence analysis) were found to be associated with vascular invasion (Iino et al, 1994), distant metastases (Kern et al, 1989; Ookawa et al, 1993; Kastrinakis et al, 1995) and shorter survival (Laurent-Puig et al, 1992; Offerhaus et al, 1992; Hamelin et al, 1994; Pricolo et al, 1996).

Within unperturbed cells, expression of wild-type (wt) p53 protein is generally below detection limits of immunohistochemistry (IHC). Stabilization of p53 protein (leading to accumulation in the nuclei) occurs after an appropriate stimulus (e.g. DNA damage), but also as a result of loss of function secondary to mutation (Blagosklonny, 1997).

*bcl*-2 is one member of a gene family, products of which are involved in either inhibition (e.g. Bcl-2, Bcl-$X_L$) or promotion of cell death (e.g. Bax, Bak). In colorectal tissues, Bcl-2 is highly expressed at the base of crypts (Hockenbery et al, 1991; Bronner et al, 1995; Merrit et al, 1995). The first studies on immunohistochemical detection of Bcl-2 in neoplasms of the colorectal tract suggested a high expression both in adenomas and carcinomas (Hague et al, 1994; Bronner et al, 1995), but more recent studies indicated a significantly lower proportion of carcinomas to be Bcl-2 positive

*Received 9 April 1997*
*Revised 8 December 1997*
*Accepted 9 December 1997*

*Correspondence to:* RAEM Tollenaar

**Table 1** bcl-2 and p53 expression and clinicopathological characteristics

| Clinicopathological variable | bcl-2 | | | p53 | | |
|---|---|---|---|---|---|---|
| | *n* | Moderate + high expression (%) | Stat | *n* | High expression, >30% pos nuclei (%) | Stat |
| Sex | | | | | | |
| Male | 113 | 17 | NS | 128 | 40 | NS |
| Female | 96 | 19 | | 110 | 41 | |
| Age | | | | | | |
| 0–50 years | 23 | 17 | NS | 25 | 32 | NS |
| >50 years | 186 | 18 | | 213 | 41 | |
| Site | | | | | | |
| Coecum–transverse colon | 54 | 13 | NS | 58 | 33 | NS |
| Splenic flexure–descending colon | 33 | 9 | | 38 | 34 | |
| Sigmoid–rectum | 122 | 22 | | 142 | 45 | |
| Differentiation | | | | | | |
| Poor | 117 | 14 | NS | 130 | 41 | NS |
| Moderate | 58 | 22 | | 67 | 40 | |
| Good | 34 | 24 | | 41 | 39 | |
| Mucinous | 57 | 18 | NS | 68 | 28 | 0.01 |
| Non-mucinous | 152 | 18 | | 170 | 45 | |
| Lymphocytic infiltration | | | | | | |
| Low | 75 | 16 | NS | 91 | 41 | NS |
| Intermediate | 94 | 22 | | 105 | 41 | |
| High | 40 | 10 | | 42 | 38 | |
| Eosinophilic infiltration | | | | | | |
| Low | 126 | 18 | NS | 141 | 44 | NS |
| Intermediate | 46 | 15 | | 54 | 41 | |
| High | 37 | 21 | | 43 | 28 | |
| Dukes' stage | | | | | | |
| A/B1 | 62 | 19 | NS | 66 | 33 | NS |
| B2 | 89 | 17 | | 104 | 42 | |
| C1+2 | 58 | 17 | | 68 | 44 | |

**Table 2** Expression of Bcl-2 in colorectal carcinomas

| SI | F | Bcl-2 score | Frequency | Percentage |
|---|---|---|---|---|
| 0 | 2[a] | 0 | 106 | 50.7 |
| 1 | 0 and 1 | 1 | 37 | 17.7 |
| 1 | 2 | 2 | 29 | 13.9 |
| 2 | 0 and 1 | 3 | 29 | 13.9 |
| 2 | 2 | 4 | 2 | 1.0 |
| 3 | 0 and 1 | 5 | 5 | 2.4 |
| 3 | 2 | 6 | 1 | 0.5 |
| | | Missing | 29 | |
| Total | | | 238 | 100.0 |

SI, staining intensity, range of scores 0–3; F, fraction of positive tumour cells, fraction of cells in each category: 0–25% = 0, 25–75% = 1, 75–100% = 2. Tumours were grouped into separate categories, not by addition or multiplication of the two scores, but by classifying them according to the fraction of cells showing the most intense staining. Tumours that had the same staining intensity (SI), but also contained a significant number of tumour cells with less intense staining (score F = 0 or F = 1) were grouped together. This scoring system resulted in Bcl-2 scores ranging from 0 to 6. [a]100% negative.

(Bosari et al, 1995; Baretton et al, 1996; Flohil et al, 1996; Watson et al, 1996; Ward et al, 1997). With respect to the prognostic significance of Bcl-2 staining in colorectal carcinomas, data were not conclusive (Bosari et al, 1995; Öfner et al, 1995; Baretton et al, 1996). Moreover, whereas in adenomas an inverse relationship was found between Bcl-2 expression and p53 accumulation, this could not be confirmed in carcinomas (Sinicrope et al, 1995; Baretton et al, 1996). However, a dual staining technique for both Bcl-2 and p53 suggested reciprocity of expression within individual tumours (Watson et al, 1996).

In the present study we evaluated IHC of both p53 and Bcl-2 in a series of 238 paraffin-embedded colorectal carcinomas from patients that had a median follow-up of 40 months. We used the monoclonal antibody (MAb) DO7 and the polyclonal antiserum CM-1 (Midgley et al, 1992) for p53 and the MAb Bcl-2:124 (Pezzella et al, 1990) for Bcl-2, analysing staining results as previously reported (Baas et al, 1994; Van Slooten et al, 1996). Our data clearly demonstrate that IHC with these antibodies does not provide information relevant to the prognosis of this series of colorectal cancer patients.

### MATERIALS AND METHODS

### Patients

From January 1980 until December 1992, 855 consecutive patients with colorectal cancer were registered at the Leiden University Hospital. Of these, 266 patients underwent curative resection for colorectal adenocarcinoma and had a post-operative follow-up. A

© Cancer Research Campaign 1998 

resection was considered curative when no tumour was left behind and the patient was alive 30 days after surgery, with no evidence of disease. These patients did not receive adjuvant chemotherapy and were followed until January 1994 or until death.

For the present study, paraffin-embedded tissue samples from 238 patients were analysed (in 20 cases tissue blocks were not available and in eight cases a double tumour was present). The pathological staging was determined using the Astler–Coller modification of Dukes' classification (Astler et al, 1954). In addition, presence of mucinous characteristics was assessed as well as the amount of lymphocytic infiltration and eosinophilic granulocyte infiltration (Jass et al, 1992). Patient and tumour characteristics are listed in Table 1.

### Immunohistochemistry

Sections (5 μm) were deparaffinized in xylene. Endogenous peroxidase was blocked by 0.3% hydrogen peroxide–methanol for 20 min. After immersing the sections in alcohol they were rehydrated. Antigen retrieval was performed by heating the sections at 100°C in 10 mM citrate buffer (pH 6.0) for 10 min. After a short rinse in phosphate-buffered saline (PBS), sections were incubated overnight at room temperature with antibodies either to p53 (clone DO-7 mouse antihuman MAb, 1:1000; CM-1 rabbit polyclonal antibody, 1:3000, Novocastra Laboratories, Newcastle upon Tyne, UK) or to Bcl-2 (clone 124 mouse antihuman MAb, 1:200, Boehringer Mannheim, Mannheim, Germany). After several washing steps in PBS, sections were incubated for 30 min with labelled second-step MAbs diluted 1:200 in PBS. For DO-7 and Bcl-2:124 we used biotinylated rabbit-anti-mouse MAbs (Dakopatts, Glostrup, Denmark); for CM-1 we used biotinylated donkey anti-rabbit anti-serum (Amersham, Cleveland, OH, USA). PBS washings were followed by incubation for 1 h with a complex of biotinylated horse radish peroxidase and streptavidin (Dakopatts), diluted 1:100 in PBS. Staining was developed in PBS containing 0.05% 3,3-diaminobenzidine tetrahydrochloride and 0.02% hydrogen peroxide. Sections were counterstained with haematoxylin, dehydrated with ethanol, cleared in xylene and mounted with malinol under a coverslip.

For p53 staining, a tumour with nuclear p53 accumulation served as a positive control. For Bcl-2 staining infiltrating lymphocytes were used as an internal positive control because it has been found that this cell population invariably contained strongly stained cells. In 29 tumours, no positive lymphocytes could be detected and these tumours were therefore not included in the Bcl-2 analysis.

### Analysis staining patterns

Nuclear staining of p53 was scored semiquantitatively by two independent observers and expressed percentage of positive tumour cells. Tumours were assigned to one of three categories: high expression, more than 30% of cancer cells stained; low expression, 1–30% of cancer cells stained; and no expression, 0% cancer cells stained (Baas et al, 1994). Apart from nuclear staining, we also estimated the presence of cytoplasmic staining; staining intensity (SI) was scored as low, intermediate or high.

Staining of Bcl-2 was evaluated simultaneously by two observers. Table 2 defines the full scoring system for the level of Bcl-2 staining. SI was scored negative (0), weakly (1), moderately (2) or strongly positive (3). Marked intratumour heterogeneity for SI was observed in one-third of cases, but only a few 'heterogeneous' tumours contained a fraction of tumour cells virtually negative for Bcl-2. For that reason it was decided not to count the number of Bcl-2-'positive' tumour cells. Instead, the fraction of cells showing the highest SI was estimated and assigned to the categories 0–25% (0), 25–75% (1) or 75–100% (2) (Van Slooten et al, 1996).

### Statistics

The relationship between the various clinicopathological parameters and p53 or Bcl-2 staining was evaluated using the chi-square test for two group comparisons. Overall survival curves were constructed according to the method of Kaplan and Meier. Survival curves were compared using the log-rank test.

### RESULTS

### p53 expression

Among the 238 adenocarcinomas studied, 77 (36.1%) were completely negative after immunostaining with DO-7, 56 (23.5%) showed ≤ 30% positive tumour cell nuclei and 96 (40.3%) showed > 30% positive nuclei. In sporadic cases we observed faint cytoplasmic p53 staining. With CM-1 immunostaining, 17 adenocarcinomas (7.1%) were completely devoid of p53 expression, whereas only 12 tumours (5.0%) showed staining confined to the nucleus. Negative nuclei were observed in 83 cases (34.9%), ≤ 30% positive nuclei in 75 cases (31.5%) and >30% positive nuclei in 80 cases (33.6%). Staining intensity (SI) of cytoplasmic staining was scored as low (42.4%), intermediate (38.7%) or high (6.7%). Only one tumour with >30% positive nuclei showed a high cytoplasmic SI. Both with DO-7 and CM-1, normal colonic mucosa was completely negative in all cases evaluated.

### BCL-2 EXPRESSION

As shown in Table 2, tumours were grouped into separate categories according to the fraction of cells showing the most intense staining; tumours with a similar SI in at least half of the tumour cell population were grouped together. Resulting categories were designated 0–6. From 219 evaluable tumours 50.7% was completely negative (0), 31.1% weakly positive (score 1+2), 14.9% moderately positive (score 3+4) and 2.9% strongly positive (score 5+6). Many tumours showed homogeneously weak staining of the carcinoma cells.

In 13 cases we found adenomatous areas at the mucosal edge of the neoplasm: in four cases Bcl-2 expression in the non-malignant cells was similar to that in their malignant counterparts, in five cases Bcl-2 was present in the non-malignant cells, but absent in the carcinoma, and in four cases both cell types were negative.

### Correlation with clinicopathological parameters, disease-free interval and survival

No correlation was found between Bcl-2 and p53 expression. The result was no different when the more detailed categorization was used for Bcl-2 or when nuclear p53 expression was analysed without the moderately stained group (1–30% positive tumour cell nuclei) or with different cut-off points between staining categories. Non-mucinous tumours showed more nuclear p53 overexpression

© Cancer Research Campaign 1998

**Figure 1** Kaplan–Meier curves of the 209 patients with colorectal carcinoma with regard to high and low Bcl-2 immunoreactivity

**Figure 2** Kaplan–Meier curves of the 238 patients with colorectal carcinoma with regard to high or low vs no nuclear p53 protein accumulation (MAb DO-7)

than mucinous tumours (MAb DO-7; $P = 0.01$). With this single exception, neither p53 nor Bcl-2 were related to any of the clinicopathological parameters (Table 1) and no significant difference in survival was detected between patients with tumour tissue displaying moderate to high vs low or no expression (Figures 1 and 2). Similar results were found for disease-free survival (data not shown). The more detailed categorization of p53 or Bcl-2 staining did not change the results and no significant correlation with disease-free or overall survival was detected for subgroups based on combined p53/Bcl-2 score (p53+/Bcl-2+; p53+/Bcl-2–; p53–/Bcl-2+; p53–/Bcl-2–; not shown).

### DISCUSSION

This study shows that IHC for p53 and Bcl-2 does not predict survival in colorectal cancer. This is in line with most of the literature. Only approximately one-third of all studies on the prognostic value of IHC of p53 in colorectal cancer indicated its potential relevance, a situation similar to that for IHC of Bcl-2 (Manne et al, 1997; Tollenaar, 1997). For instance, Manne et al (1997) recently concluded from a series of 134 patients that both parameters independently give relevant information on prognosis. Data from their study confirm our own conclusion that IHC of p53 does not seem to correlate with clinicopathological parameters, except for the more prominent presence of p53 in non-mucinous tumours; the latter phenomenon was also noticed by other investigators (Campo et al, 1991; Hanski et al, 1992; Mulder et al, 1995) and adds to the likelihood that mucus-producing tumours (being approximately 10–15% of colorectal carcinomas) constitute a subset of colorectal neoplasms with a different biology. But for both p53 and Bcl-2 the conclusions differ with respect to patient survival, and it is difficult to establish whether this results from differences in materials and methods or from differences in patient- (and tumour)-related characteristics. It might be relevant in this context that the patient groups differed in age distribution, ethnic (i.e. genetic) background, ratio of left- and right-sided tumours, presence of distant metastases and whether or not adjuvant chemotherapy had been given.

Technical differences are seen in the type of antibodies and antigen retrieval methods used as well as the percentage of p53-positive cells used as cut-off point. Horne et al (1996) recently concluded from a panel of MAbs used on breast cancers that pAb1801 and DO-7 are among the most effective antibodies for IHC in routinely processed material. A similar study on colon cancers revealed that staining with DO-7 after an antigen retrieval method for paraffin sections was the most sensitive and specific procedure (Baas et al, 1994). It is therefore remarkable that Manne et al (1997), who used BP53-12-1 MAb after having established that it gave similar results as pAb1801, reported that the above antigen retrieval procedure abolished the relationship found between p53 positivity and patient survival.

The polyclonal antiserum CM-1 was reported to recognize both mutant and wt-p53 on formalin-fixed material, and its use resulted in both nuclear and cytoplasmic staining patterns (Midgley et al, 1992). In colorectal cancers, one study found nuclear CM-1 staining to be correlated with unfavourable clinical outcome (Starzynska et al, 1992), but the follow-up period was short and studies with longer follow-ups did not confirm this result (Sun et al, 1992; Bosari et al, 1994; Mulder et al, 1995). Two reports described the presence of marked cytoplasmic staining and provided data suggesting that, whereas nuclear staining was not an independent prognostic factor, cytoplasmic staining was highly significantly associated with poor survival (Sun et al, 1992; Bosari et al, 1994) However, this remarkable finding was not confirmed by the present study.

Our data on Bcl-2 positivity are in line with published results from relatively large patient series (Bosari et al, 1995; Öfner et al, 1995; Manne et al, 1997), showing no staining (except in infiltrating lymphocytes) in about half of the tumours and strong staining in a small subpopulation. We were not able to detect a significant correlation between Bcl-2 expression and any of the clinicopathological parameters evaluated, confirming data obtained by Bosari et al (1995). Reported correlations include tumour differentiation (Watson et al, 1996; Schneider et al, 1997), lymphocyte infiltration and tumour size (Öfner et al, 1995), and factors such as bowel wall and regional lymph node invasion and presence of distant metastases (Manne et al, 1997). It was also reported that Bcl-2-negative tumours tend to have higher apoptotic indices (Baretton et al, 1996). But, as with p53, the literature is far from equivocal on this subject.

In multivariate analysis, two research groups found Bcl-2 to be an independent prognostic parameter with Dukes' classification or

© Cancer Research Campaign 1998

TNM as stratification factor (Öffner et al, 1995; Manne et al, 1997), but others could not confirm this independence (Baretton et al, 1996) and neither we nor Bosari et al (1993) could find any correlation with a better clinical course. Moreover, using a MAb different from that used in the above studies, Schneider et al (1997) concluded Bcl-2 expression not to be associated with survival or response to 5-fluorouracil-based chemotherapy.

Based on observed abnormal Bcl-2 immunoreactivity in the earliest precursor dysplastic lesions and in contiguous non-dysplastic epithelium, it has been proposed that Bcl-2 alterations may occur very early in the sequence of events leading to gastrointestinal neoplasia (Bronner et al, 1995). Within normal mucosal tissue adjacent to the carcinomas, we detected Bcl-2 overexpression in basal and middle parts of the crypts – the more differentiated surface epithelial cells were invariably negative. Adenomatous areas showed either a similar staining pattern to the carcinoma cells or a higher expression. Because a majority of studies reported a higher frequency of Bcl-2-positive adenomas compared with carcinomas, this would suggest that, although many adenomas are derived from cells expressing relatively high levels of Bcl-2, during progression towards overt carcinoma the expansion of cell clones with lower (or even no) expression of Bcl-2 is strongly favoured. Moreover, it has been reported that colon carcinomas that have no adenomatous elements in their vicinity ('de novo' carcinomas) tend to have even lower Bcl-2 expression than 'ex-adenoma' carcinomas (Mueller et al, 1996).

Using a dual-staining technique, Watson et al (1996) noticed in both adenomas and carcinomas reciprocity of expression of Bcl-2 and p53, and conjectured that accumulation of mutant p53 could possibly silence the *bcl*-2 gene. However, other investigators found reciprocity in adenomas, but not in carcinomas (Sinicrope et al, 1995; Baretton et al, 1996) and, although we and others have found a strong reciprocity of Bcl-2 and p53 in breast cancers (Van Slooten et al, 1996), this reciprocity was virtually absent in the present series. Also, Mosnier et al (1996) recently concluded that in colorectal cancers deregulation of *bcl*-2 is probably not dependent on p53 gene mutations.

A more likely explanation for loss of Bcl-2 is based on the increasing evidence that Bcl-2 not only reduces apoptosis, but also affects the cell cycle machinery (Watson et al, 1996). We and others noticed that in breast cancers a strong inverse correlation exists between Bcl-2 expression and proliferative activity (Van Slooten et al, 1996) and Pietenpol et al (1994) described that in several tumour cells lines (including colorectal cancer cells) overexpression of Bcl-2 resulted in severe growth inhibition. More recently, a number of reports have provided evidence that Bcl-2 can inhibit the transition of cells from a resting to a cycling phase and stimulate the reverse condition (Mazel et al, 1996; Huang et al, 1997). However, it is not clear yet to what extent the loss of apoptosis-inhibiting inhibiting function of Bcl-2 is compensated by modulation of other factors involved in apoptosis regulation – including the pro-apoptotic members of the Bcl-2 family (Krajewska et al, 1996; Rampino et al, 1997).

Our results demonstrate that, even in a carefully selected series of patients who underwent curative surgery without adjuvant chemotherapy, p53 and Bcl-2 used as immunohistochemical markers do not seem to have the potential to improve individually tailored prognostication, and therefore we have little reason to share the optimism expressed by some other investigators. The only way to establish whether this discrepancy in findings is merely based on technical aspects would be to exchange the paraffin-embedded tumour material between the different research groups. It might well be possible, however, that various tumour-related factors may profoundly affect the impact of p53 and Bcl-2 immunostaining patterns on prognosis and that more information about these factors is needed to identify reliably relevant subtypes of colorectal cancer.

### ACKNOWLEDGEMENT

We would like to thank Rob Keijzer for his technical assistance.

### REFERENCES

Astler VB and Coller FA (1954) The prognostic significance of direct extension of carcinoma of the colon and rectum. *Ann Surg* **139**: 846–852

Baas IO, Mulder J-WR, Offerhaus GJA, Vogelstein B and Hamilton SR (1994) An evaluation of six antibodies for immunohistochemistry of mutant p53 gene product in archival colorectal neoplasms. *J Pathol* **172**: 5–12

Baker SJ, Preisinger AC, Jessup M, Paraskeva C, Markowitz S, Willson JKV, Hamilton S and Vogelstein B (1990) p53 gene mutations occur in combination with 17p allelic deletions as late events in colorectal tumorigenesis. *Cancer Res* **50**: 7717–7722

Baretton GB, Diebold J, Christoforis G, Vogt M, Müller C, Dopfer K, Schneiderbanger K, Schmidt M and Löhrs U (1996) Apoptosis and immunohistochemical bcl-2 expression in colorectal adenomas and carcinomas; aspects of carcinogenesis and prognostic significance. *Cancer* **77**: 255–264

Bedi A, Pasricha PJ, Akhtar AJ, Barber JP, Bedi GC, Giardiello FM, Zehnbauer BA, Hamilton SR and Jones RJ (1995) Inhibition of apoptosis during development of colorectal cancer. *Cancer Res* **55**: 1811–1816

Blagosklonny MV (1997) Loss of function and p53 stabilization. *Oncogene* **15**: 1889–1893

Bosari S, Viale G, Bossi P, Maggioni M, Coggi G, Murray JJ and Lee KC (1994) Cytoplasmic accumulation of p53 protein: an independent prognostic indicator in colorectal adenocarcinomas. *J Natl Cancer Inst* **86**: 681–687

Bosari S, Moneghini L, Graziani D, Lee AKC, Murray JJ, Coggi G and Viale G (1995) Bcl-2 oncoprotein in colorectal hyperplastic polyps, adenomas, and adenocarcinomas. *Human Pathol* **26**: 534–540

Bronner MP, Culin C, Ree JC and Furth EE (1995) The bcl-2 proto-oncogene and the gastrointestinal epithelial tumor progression model. *Am J Pathol* **146**: 20–26

Campo E, de la Calle-Martin O, Miquel R, Palacin A, Romero M, Fabregat V, Vives J, Cardesa A and Yague J (1991) Loss of heterozygosity of p53 gene and p53 protein expression in human colorectal carcinomas. *Cancer Res* **51**: 4436–4442

Fearon ER and Vogelstein B (1990) A genetic model for colorectal tumorigenesis. *Cell* **61**: 759–767

Flohil CC, Janssen PA and Bosman FT (1996) Expression of Bcl-2 protein in hyperplastic polyps, adenomas, and carcinomas of the colon. *J Pathol* **178**: 393–397

Hague A, Moorghen M, Hicks D, Chapman M and Paraskeva C (1994) BCL-2 expression in human colorectal adenomas and carcinomas. *Oncogene* **9**: 3367–3370

Hamelin R, Laurent-Puig P, Olschwang S, Jego N, Asselain B, Remvikos Y, Girodet J, Salmon RJ and Thomas G (1994) Association of p53 mutations with short survival in colorectal cancer. *Gastroenterology* **106**: 42–48

Hanski C, Bornhoeft G, Shimoda T, Hanski M-L, Lane DP, Stein H and Riecken E-O (1992) Expression of p53 protein in invasive colorectal carcinomas of different histological types. *Cancer* **70**: 2772–2777

Hockenbery DM, Zutter M, Hickey W, Nahm M and Korsmeyer SJ (1991) Bcl-2 protein is topographically restricted in tissues characterized by apoptotic cell death. *Proc Natl Acad Sci USA* **88**: 6961–6965

Hollstein M, Sidransky D, Vogelstein B and Harris CC (1991) P53 mutations in human cancers. *Science* **253**: 49–53

Horne GM, Anderson JJ, Tiniakos DG, McIntosh GG, Thomas MD, Angus B, Henry JA, Lennard TWJ and Horne CWH (1996) P53 protein as a prognostic indicator in breast carcinoma: a comparison of four antibodies for immunohistochemistry. *Br J Cancer* **73**: 29–35

Huang DCS, O'Reilly LA, Strasser A and Cory S (1997) The anti-apoptosis function of Bcl-2 can be genetically separated from its inhibitory effect on cell cycle entry. *EMBO J* **16**: 4628–4638

Iino H, Fukayama M, Maeda Y, Koike M, Mori T, Takahashi T, Kikuchi-Yanoshita R, Miyaki M, Mizuno S and Watanabe S (1994) Molecular genetics for clinical management of colorectal cancer. *Cancer* **73**: 1324–1331

 © Cancer Research Campaign 1998

Jass JR (1992) Lymphocytic infiltration and survival in rectal cancer. *J Clin Pathol*, **39**, 585–589

Kaklamanis L, Gatter KC, Mortensen N, Baigrie RJ, Heryet A, Lane DP and Harris AL (1993) p53 expression in colorectal adenomas. *Am J Pathol* **142**: 87–93

Kastrinakis WV, Ramchurren N, Rieger KM, Hess DT, Loda M, Steele G and Summerhayes IC (1995) Increased incidence of p53 mutations is associated with hepatic metastasis in colorectal neoplastic progression. *Oncogene* **11**: 647–652

Kern SE, Fearon ER, Tersmette KWF, Enterline JP, Leppert M, Nakamura Y, White R, Vogelstein B and Hamilton SR (1989) Allelic loss in colorectal carcinoma. *JAMA* **261**: 3099–3103

Kikuchi-Yanoshita R, Konishi M, Ito S, Seki M, Tanaka K, Maeda Y, Iino H, Fukayama M, Koike M, Mori T, Sakuraba H, Fukunari H, Iwama T and Miyaki M (1992) Genetic changes of both p53 alleles associated with the conversion from colorectal adenoma to early carcinoma in familial adenomatous polyposis and non-familial adenomatous polyposis patients. *Cancer Res* **52**: 3965–3971

Krajewska M, Moss SF, Krajewski S, Song K, Holt PR and Reed JC (1996) Elevated expression of Bcl-X and reduced Bak in primary colorectal adenocarcinomas. *Cancer Res* **56**: 2422–2427

Laurent-Puig P, Olschwang S, Remvikos Y, Asselain B, Melot T, Validire P, Muleris M, Girodet J, Salmon RJ and Thomas G (1992) Survival and acquired genetic alterations in colorectal cancer. *Gastroenterology* **102**: 1136

Manne U, Myers RB, Moron C, Poczatek RB, Dillard S, Weiss H, Brown D, Srivastava S and Grizzle WE (1997) Prognostic significance of Bcl-2 expression and p53 nuclear accumulation in colorectal adenocarcinoma. *Int J Cancer* **74**: 346–358

Mazel S, Burtrum and Petrie HT (1996) Regulation of cell division cycle progression by bcl-2 expression: a potential mechanism for inhibition of programmed cell death. *J Exp Med* **183**: 2219–2226

Merrit AJ, Potten CS, Watson AJM, Loh DY, Nakayama K-I, Nakayama K and Hickman JA (1995) Differential expression of bcl-2 in intestinal epithelia; correlation with attenuation of apoptosis in colonic crypts and the incidence of colonic neoplasia. *J Cell Science* **108**: 2261–2271

Midgley CA, Fisher CJ, Bartek J, Vojtesek B, Lane D and Barnes DM (1992) Analysis of p53 expression in human tumours: an antibody raised against human p53 expressed in *Escherichia coli*. *J Cell Sci* **101**: 183–189

Mosnier J-F, Perret AG, Vindimian M, Dumollard JM, Balique JG, Perpoint B and Boucheron S (1996) An immunohistochemical study of the simultaneous expression of bcl-2 and p53 oncoproteins in epithelial tumors of the colon and rectum. *Arch Pathol Lab Med* **120**: 654–659

Mueller J, Mueller E, Hoepner I, Jütting J, Bethke B, Stolte M and Höfler H (1996) Expression of bcl-2 and p53 in de novo and ex-adenoma colon carcinoma: a comparative immunohistochemical study. *J Pathol* **180**: 259–265

Mulder J-WR, Baas IO, Polak MM, Goodman SN and Offerhaus GJA (1995) Evaluation of p53 protein expression as a marker for long-term prognosis in colorectal carcinoma. *Br J Cancer* **71**: 1257–1262

Offerhaus GJA, de Feyter EP, Cornelisse CJ, Tersmette KWF, Fo J, Kern SE, Vogelstein B and Hamilton SR (1992) The relationship of DNA aneuploidy to molecular genetic alterations in colorectal carcinoma. *Gastroenterology* **102**: 1612–1619

Öfner D, Riehemann K, Maier H, Riedmann B, Nehoda H, Tötsch M, Böcker W, Jasani B and Schmid KW (1995) Immunohistochemically detectable bcl-2 expression in colorectal carcinoma: correlation with tumour stage and patient survival. *Br J Cancer* **72**: 981–985

Ookawa K, Sakamoto M, Hirohashi S, Yoshida Y, Sugimura T, Terada M and Yokota J (1993) Concordant p53 and DCC alterations and allelic losses on chromosome 13q and 14q associated with liver metastases of colorectal carcinoma. *Int J Cancer* **53**: 382–387

Pezzella F, Tse AGD, Cordell JL, Pulford KAF, Gatter KC and Mason DY (1990) Expression of the bcl-2 oncogene protein is not specific for the 14;18 chromosomal translocation. *Am J Pathol* **137**: 225–232

Pietenpol JA, Papadopoulos N, Markowitz S, Willson JK, Kinzler KW and Vogelstein B (1994) Paradoxical inhibition of solid tumor cell growth by bcl2. *Cancer Res* **54**: 3714–3717

Pricolo VE, Finkelstein SD, Wu T-T, Keller G, Bakker A, Swalsky PA and Bland KI (1996) Prognostic value of TP53 and K-ras-3 mutational analysis in stage III carcinoma of colon. *Am J Surg* **171**: 41–46

Rampino M, Yamamoto H, Ionov Y, Li Y, Sawai M, Reed JC and Perucho M (1997) Somatic mutations in the BAX gene in colon cancers of the microsatellite mutator phenotype. *Science* **275**: 967–969

Schneider HJ, Sampson SA, Cunningham D, Norman AR, Andreyev HJN, Tilsed JVT and Clarke PA (1997) Bcl-2 expression and response to chemotherapy in colorectal adenocarcinomas. *Br J Cancer* **75**: 427–431

Sinicrope FA, Ruan SB, Cleary KR, Stephens LC, Lee JJ and Levin B (1995) bcl-2 and p53 oncogene expression during colorectal tumorigenesis. *Cancer Res* **55**: 237–241

Starzynska T, Bromley M, Ghosh A and Stern PL (1992) Prognostic significance of p53 overexpression in gastric and colorectal carcinoma. *Br J Cancer* **66**: 558–562

Sun X-F, Carstensen JM, Zhang H, Stal O, Wingren S, Hatschek T and Nordenskjöld B (1992) Prognostic significance of cytoplasmic p53 oncoprotein in colorectal adenocarcinoma. *Lancet* **340**: 1369–1373

Tollenaar RAEM (1997) Aspects of tumour progression in colorectal carcinoma. Thesis, Leiden, The Netherlands

Van Slooten H-J, Clahsen PC, van Dierendonck JH, Duval C, Pallud C, Mandard A-M, Delobelle-Deroide A, van de Velde CJH and Van De Vijver MJ (1996) Expression of BCL-2 in node-negative breast cancer is associated with various prognostic factors, but does not predict response to one course of perioperative chemotherapy. *Br J Cancer* **74**: 78–85

Ward RL, Todd AV, Santiago F, O'Connor T and Hawkins NJ (1997) Activation of the K-ras oncogene in colorectal neoplasms is associated with decreased apoptosis. *Cancer* **79**, 1106–1113

Watson AJM, Merrit AJ, Jones LS, Askew JN, Anderson E, Becciolini A, Balzi M, Potten CS and Hickman JA (1996) Evidence of reciprocity of bcl-2 and p53 expression in human colorectal adenomas and carcinomas. *Br J Cancer* **73**: 889–895

© Cancer Research Campaign 1998